# Pain severity and analgesics use in the community-dwelling older population: a drug utilization study from Germany

**DOI:** 10.1007/s00228-020-02954-5

**Published:** 2020-07-10

**Authors:** Thi Ngoc Mai Nguyen, Dana Clarissa Laetsch, Li-Ju Chen, Walter Emil Haefeli, Andreas D Meid, Hermann Brenner, Ben Schöttker

**Affiliations:** 1 Division of Clinical Epidemiology and Aging Research, German Cancer Research Center (DKFZ), Heidelberg, Germany; 2 Network Aging Research, University of Heidelberg, Heidelberg, Germany; 3 Department of Clinical Pharmacology and Pharmacoepidemiology, Heidelberg University Hospital, Heidelberg, Germany

**Keywords:** Analgesics, Opioids, Metamizole, NSAIDs, Drug utilization study, Older adult

## Abstract

**Purpose:**

Chronic pain is common in the older population and a significant public health concern. However, comprehensive studies on analgesics use in this age group from Germany are scarce. This study aims to give a comprehensive overview on the use of the most common therapeutic groups of analgesics in community-dwelling older adults from Germany.

**Methods:**

A cross-sectional study was carried out using data from a German cohort of 2038 community-dwelling adults aged 63–89 years. Descriptive statistics and logistic regression models were applied to assess the utilization of analgesics by age, sex, pain severity, pain duration, and locations.

**Results:**

One out of four study participants was suffering from high-intensity or disabling pain. Approximately half of those taking analgesics still reported to suffer from high-intensity or disabling pain. Among analgesics users, occasional non-steroidal anti-inflammatory drugs (NSAIDs) use was the most frequent pain therapy (in 43.6% of users), followed by metamizole (dipyrone) use (16.1%), regular NSAIDs use (12.9%), strong opioids use (12.7%), and weak opioids use (12.0%). In multivariate logistic regression models, higher age, higher pain severity, longer pain duration, abdominal pain, and back pain were statistically significantly associated with opioids use. Metamizole use was also statistically significantly associated with higher pain severity but inversely associated with pain duration.

**Conclusions:**

A significant number of older German adults are affected by high-intensity and disabling chronic pain despite receiving analgesics. Long-term studies are needed to compare the effectiveness and safety of different treatments for chronic pain in older adults.

**Electronic supplementary material:**

The online version of this article (10.1007/s00228-020-02954-5) contains supplementary material, which is available to authorized users.

## Introduction

Pain is one of the most common reasons for seeking medical care [1]. Chronic pain is defined as pain that lasts or recurs for longer than 3 months [2], and interferes significantly with the daily life of people affected. The European Pain Federation declared that while “acute pain may reasonably be considered a symptom of disease or injury, chronic and recurrent pain is a specific healthcare problem, a disease in its own right” [3]. In a survey from Germany, approx. 28% of older adults were found to be suffering from pain in the last 3 months and many had disabling pain [4]. A cross-European survey confirmed that one out of five European adults suffers from moderate to severe chronic pain, and among those, one-third are not being treated [5].

Managing chronic pain is challenging. Although most analgesics are considered safe when used intermittently at recommended doses, they pose potential hazards under prolonged use, particularly in older populations [6, 7]. NSAIDs use can cause or exacerbate cardiovascular disease, chronic kidney disease, and peptic ulcer disease. Adverse effects associated with opioids, such as constipation, respiratory depression, and addiction, can present a barrier to long-term treatment [8]. In recent years, the topic of analgesics use has received much attention in Europe [9–14]. However, the existing drug utilization studies, done on analgesics use in the older German population, either include only nursing home residents [15, 16] or multi-morbid older adults [17], or focus on selected analgesics [13, 18].

This study aims to give a comprehensive overview on the prevalence of overall analgesics use and the prevalence of use of the most common therapeutic groups of analgesics in community-dwelling older adults from Germany. Overall prevalence is further stratified by age, sex, pain severity, and pain duration. Multivariate logistic regression models are applied to identify the determinants of opioids and metamizole use.

## Methods

### Study design and data collection

We conducted a cross-sectional survey using the 14-year follow-up data from the ESTHER study (German name: Epidemiologische **S**tudie zu Chancen der Verhütung, Früherkennung und optimierten Therapie chronischer Erkrankungen in der älteren Bevölkerung). Details of the study design have been reported elsewhere [19, 20]. In brief, ESTHER is an ongoing, population-based cohort study from Saarland, Germany. Between July 2000 and December 2002, 9940 individuals, aged 50–75 years, were recruited via their general practitioners (GPs) during a routine health check-up. In total, 420 GPs were recruited for the study [19]. After 2, 5, 8, 11, and 14 years, participants were contacted again and asked to complete a questionnaire to provide information on their current health status and medication use. In the 8-, 11-, and 14-year follow-up, participants were additionally offered a home visit by a study physician who recorded all regularly used drugs that the participants had at home and conducted geriatric assessments. At the time of the 14-year follow-up, *n* = 2104 had died, *n* = 3897 completed the participants’ questionnaire, and *n* = 2143 took part in the home visit. In order to use the most up to date data in a population with advanced age, we included those 2038 study participants in this survey who completed the medication evaluation at the 14-year follow-up home visit, which was conducted from September 2014 to October 2016.

Age and sex were collected from the participants’ questionnaires. Information on medical history was gathered by asking participants as well as their GPs in questionnaires about specific diagnoses. This article was written in adherence to the STROBE checklist for reporting of observational study results.

### Assessment of pain

The evaluation of pain severity and duration was based on questions from the German Pain Questionnaire (Deutscher Schmerz-Fragebogen-DSF) [21]. It includes a German adaption of the Chronic Pain Grade Scale [22], designed to evaluate overall chronic pain based on two dimensions: pain intensity and pain-related disability. These two dimensions were then combined to classify chronic pain into five hierarchical categories: no pain (grade 0), low intensity and low disability (grade I), high intensity and low disability (grade II), high disability and moderately limiting (grade III), high disability and severely limiting (grade IV) [22]. For duration of pain, the question was “How long has the pain existed?” The answers were combined to for less than 6 months/6 months to 2 years/2 to 5 years/more than 5 years for descriptive analysis, and were combined further to for less than 2 years/2 to 5 years and more than 5 years for logistic regression analysis.

Locations of pain (abdominal, back, head, limbs, or joints) were extracted from the physical symptoms questionnaire of the Patient Health Questionnaire (PHQ-15) [23]. The question was “During the past four weeks, how much have you been bothered by any of those problems: Abdominal pain/ back pain/ headache/ limbs and joints pain? – Not bothered at all/ Bothered a little/ Bothered a lot.” The latter two categories were combined to obtain dichotomous variables.

### Assessment of medications

Drug data were obtained according to the “Brown Bag” method. During the home visit, a study physician encouraged study participants to show all the currently taken medicines they had at home, including over-the-counter (OTC) drugs. Information on doses and the pattern of intake (as-needed/regularly) were also documented. For this study, Anatomical Therapeutic Chemical Classification (ATC) [24] codes were used to allocate analgesics user to the following 7 therapeutic groups: strong opioids user, weak opioids user, metamizole user, regular nonsteroidal anti-inflammatory drugs (NSAIDs) user, occasional NSAIDs user, user of other analgesics (incl. acetaminophen and anti-migraine preparations), and user of adjuvants (antidepressants and anticonvulsants) (see Table S1, Online Resources 1, for ATC codes). Metamizole was considered as its own therapeutic group because it is a first-line non-opioid analgesics drug in Germany and very frequently used. If a patient used more than one analgesic, the drug with the highest analgesics potency determined which group of analgesics users the patient was allocated to. We consulted the WHO Analgesic Ladder, BG Well’s Pharmacotherapy Handbook, and a Cochrane Review about metamizole to rank substance groups according to their analgesic potency [25–27]. Adjuvants alone were not considered a separate group when patients were allocated to exclusive therapeutic groups.

The following substances were not considered to identify chronic users of analgesics (ATC code(s)): acetylsalicylic acid with a daily dose of 325 mg or lower (B01AC06), topical formulations of analgesics (M02), specific antirheumatic agents (M01C), homeopathic or anthroposophical analgesics (M01BH, N02BH), herbal analgesics (M01BP, N02BP), muscle relaxants (M03), anesthetics (N01), preparations used against cough and colds (R05), and antispasmodics (A03C, A03E).

### Statistical methods

To describe the study population, the following variables were displayed as proportions in appropriate groups: age, sex, comorbidities, pain locations, pain severity, pain duration, and analgesics drug utilization (overall and subdivided into therapeutic groups). Differences between analgesics users and non-users were identified using Chi-squared tests.

The prevalence of the use of therapeutic analgesics groups was stratified by age groups (5-year intervals), sex, pain severity (grades I–IV), and pain locations. Logistic regression was used to assess the associations of age, sex, pain severity, pain duration, and pain locations with the use of opioids and metamizole in two distinct multivariate models. In the model for opioids, users of strong and weak opioid were combined as cases. The users of other analgesic drugs were used as controls. In the metamizole model, opioids users were excluded. Metamizole users were treated as cases and compared with users of other analgesic drugs with lower analgesic potency as controls.

Two-sided *p* values less than 0.05 were considered to indicate statistical significance. All analyses were performed using SAS version 9.4 (SAS Institute, Cary, NC). Microsoft Excel 2016 was used for the generation of graphs.

## Results

### Characteristics of analgesics users and non-users

In total, 2143 people participated in the home visit at 14-year follow-up. Of these, 105 did not complete the medical assessment, leading to the final sample size of 2038. Characteristics of the study population are shown in Table 1. The mean age (standard deviation, SD) was 74.5 (6.1) years, ranging from 63 to 89 years. Approximately half of the participants were female (53.3%). About half of the study participants felt affected by pain in the back (52.0%) and in the limbs or joints (56.0%), respectively, while less felt affected by abdominal pain (15.8%) or headache (15.8%). Also, 53.1% of the participants reported having experienced pain of at least low intensity accompanied by low disability in the last 4 weeks (grades I–IV). Of those, 12.5% suffered from pain for less than 6 months, and the majority (51.8%) had chronic pain for more than 5 years. One-fourth (25.0%) were suffering from high-intensity or disabling pain (grades II–IV).

**Table 1:** Characteristics of the study population, divided into analgesics users and non-users

	All participants (*n* = 2038)	Analgesics users (*n* = 466)	Non-users (*n* = 1572)	*p* value
Age, mean (SD)	74.5 (6.1)	74.7 (6.2)	74.4 (6.1)	0.43
Age groups, *n* (%)				0.29
63–69 years	527 (25.9)	116 (24.9)	411 (26.2)	
70–74 years	441 (21.6)	100 (21.5)	341 (21.7)	
75–79 years	645 (31.7)	156 (33.5)	489 (31.1)	
80–84 years	306 (15.0)	60 (12.9)	246 (15.7)	
85–89 years	119 (5.8)	34 (7.3)	85 (5.4)	
Sex, *n* (%)				*< 0.01*
Male	951 (46.7)	*190 (40.8)*	*761 (48.4)*	
Female	1087 (53.3)	*276 (59.2)*	*811 (51.6)*	
Comorbidities, *n* (%)				
Cancer ^a^	124 (6.1)	25 (5.4)	99 (6.3)	0.58
Cardiovascular disease ^b^	730 (35.8)	183 (39.3)	547 (34.8)	0.08
Peripheral vascular disease	431 (21.2)	*133 (28.5)*	*298 (19.0)*	*< 0.01*
Diabetes mellitus type 2	546 (26.8)	*142 (30.5)*	*404 (25.7)*	*0.04*
Peptic ulcer disease	309 (15.2)	*90 (19.3)*	*219 (13.9)*	*< 0.01*
Depression	575 (28.2)	*170 (36.5)*	*405 (25.8)*	*< 0.01*
Gout	398 (19.5)	*106 (22.8)*	*292 (18.6)*	*0.046*
Arthrosis/arthritis	604 (29.9)	*204 (44.4)*	*400 (25.6)*	*< 0.01*
Joint replacement	369 (18.1)	*128 (27.5)*	*241 (15.3)*	*< 0.01*
Pain locations, *n* (%)				
Back	1037 (52.0)	*346 (76.0)*	*691 (44.9)*	*< 0.01*
Limbs and joints	1121 (56.0)	*346 (75.7)*	*775 (50.1)*	*< 0.01*
Abdominal	316 (15.8)	*99 (21.5)*	*217 (14.1)*	*< 0.01*
Head	316 (15.8)	*113 (24.8)*	*203 (13.2)*	*< 0.01*
Pain severity, *n* (%)				*< 0.01*
0	937 (46.9)	*65 (14.5)*	*872 (56.3)*	
I	561 (28.1)	*157 (35.0)*	*404 (26.1)*	
II	247 (12.4)	*106 (23.7)*	*141 (9.1)*	
III	170 (8.5)	*73 (16.3)*	*97 (6.3)*	
IV	83 (4.1)	*47 (10.5)*	*36 (2.2)*	
Pain duration among patients with pain severity grades I–IV ^c^, *n* (%)				0.23
Less than 6 months	130 (12.5)	43 (11.4)	87 (13.1)	
6 months to 2 years	183 (17.6)	55 (14.6)	128 (19.3)	
2 to 5 years	188 (18.1)	71 (18.8)	117 (17.6)	
More than 5 years	539 (51.8)	208 (55.2)	331 (49.9)	
Analgesics use, *n* (%)				
Strong opioids ^d^	59 (2.9)	59 (12.7)	0 (0)	
Weak opioids ^e^	60 (2.9)	60 (12.9)	0 (0)	
Metamizole	103 (5.1)	103 (22.1)	0 (0)	
NSAIDs, regular use	74 (3.6)	74 (15.9)	0 (0)	
NSAIDs, occasional use	222 (10.9)	222 (47.6)	0 (0)	
Adjuvant analgesics ^f^	51 (2.5)	51 (10.9)	0 (0)	
Others ^g^	26 (1.3)	26 (5.6)	0 (0)	

Statistically significant results are in italics

*SD* standard deviation, *NSAIDs* nonsteroidal anti-inflammatory drugs

^a^Diagnosed within the last 5 years

^b^Stroke, myocardial infarction, coronary heart disease, had undergone bypass surgery

^c^Analysis done in *n* = 1040 patients with pain severity grades I–IV and without missing information for pain duration. Of these patients, 377 patients were analgesics users and 663 study participants were non-users of analgesics

^d^Fentanyl, hydromorphone, morphine, oxycodone, tapentadol, buprenorphine

^e^Codeine, tilidine, tramadol, dihydrocodeine

^f^Amitriptyline, clomipramine, imipramine, trimipramine, duloxetine, fluoxetine, gabapentin, pregabalin, carbamazepine used in combination with other analgesics

^g^Acetaminophen, other anilides, antimigraine preparations, antispasmodics in combination with analgesics

Overall, 466 study participants (22.9%) used at least one analgesics drug. Analgesics users were more likely to be female, and except for cancer and cardiovascular diseases, presented with more comorbidities (Table 1). The two groups did not differ statistically significantly by age. Analgesics users reported more frequently to be bothered by back pain, pain in the limbs or joints, abdominal pain and headache, and had longer pain duration (though not statistically significantly) and had higher pain severity. High-intensity or disabling pain (grades II–IV) was present in 50.5% of the treated study participants and, at the same time, in 17.6% of the non-treated patients.

Among the 466 analgesics users, occasional NSAIDs use was the most frequent (47.6%), followed by metamizole (22.1%), regular NSAIDs (15.9%), weak opioids (12.9%), strong opioids (12.7%), adjuvant analgesics (10.9%), and others (incl. acetaminophen) (5.6%).

Overall, *n* = 363 (77.9%) had monotherapy. Furthermore, *n* = 84 (18.0%), *n* = 18 (3.9%), and *n* = 1 (0.2%) used combinations of 2, 3, and 4 analgesics, respectively. Table 2 shows the prevalence of monotherapy and combination therapies of different therapeutic analgesics groups. The majority of participants who used weak opioids, NSAIDs regularly, or occasionally used those as monotherapy (70.0%, 74.3%, and 82.9%, respectively). Users of strong opioids frequently also used metamizole (37.3%). Adjuvant analgesics users most frequently also used metamizole (41.2%), followed by combinations with strong opioids (33.3%) and occasional NSAIDs (29.4%) use. Users of other analgesics, such as acetaminophen, frequently also used NSAIDs occasionally (34.6%).

**Table 2:** Prevalence of monotherapy and combination therapy of different therapeutic analgesics groups

Therapeutic analgesics groups	Monotherapy (*n* (%))	Combinations with other therapeutic analgesics groups						
		Strong opioids (*n* (%))^a^	Weak opioids (*n* (%))^a^	Metamizole (*n* (%))^a^	NSAIDs, regular use (*n* (%))^a^	NSAIDs, occasional use (*n* (%))^a^	Adjuvant analgesics (*n* (%))^a^	Others (*n* (%))^a^
Strong opioids	20 (33.9)	N.A.	0 (0.0)	22 (37.3)	5 (8.5)	6 (10.2)	17 (28.8)	2 (3.4)
Weak opioids	42 (70.0)	0 (0.0)	N.A.	6 (10.0)	4 (6.7)	3 (5.0)	8 (13.3)	1 (1.7)
Metamizole	50 (48.5)	22 (21.4)	6 (5.8)	N.A.	6 (5.8)	12 (11.7)	21 (20.4)	2 (1.9)
NSAIDs, regular use	55 (74.3)	5 (6.8)	4 (5.4)	6 (8.1)	N.A.	0 (0.0)	7 (9.5)	1 (1.4)
NSAIDs, occasional use	184 (82.9)	6 (2.7)	3 (1.4)	12 (5.4)	0 (0.0)	N.A.	15 (6.8)	9 (4.1)
Adjuvant analgesics	0 ^b^ (0.0)	17 (33.3)	8 (15.7)	21 (41.2)	7 (13.7)	15 (29.4)	N.A.	0 (0.0)
Others	12 (46.2)	2 (7.7)	1 (3.8)	2 (7.7)	1 (3.8)	9 (34.6)	0 (0.0)	N.A.

*N.A.*, not applicable

^a^The total percent was calculated by row with the number of users of the specific therapeutic group as the denominator. For each row, the sum of percentages can exceed 100% due to 19 study participants, who had a combination of 3 or 4 analgesics and were counted more than once

^b^Per definition, adjuvant analgesics were only counted when added to another analgesic because we did not record the indications of drug prescriptions

### The utilization of different therapeutic analgesic groups

**Fig. 1:**
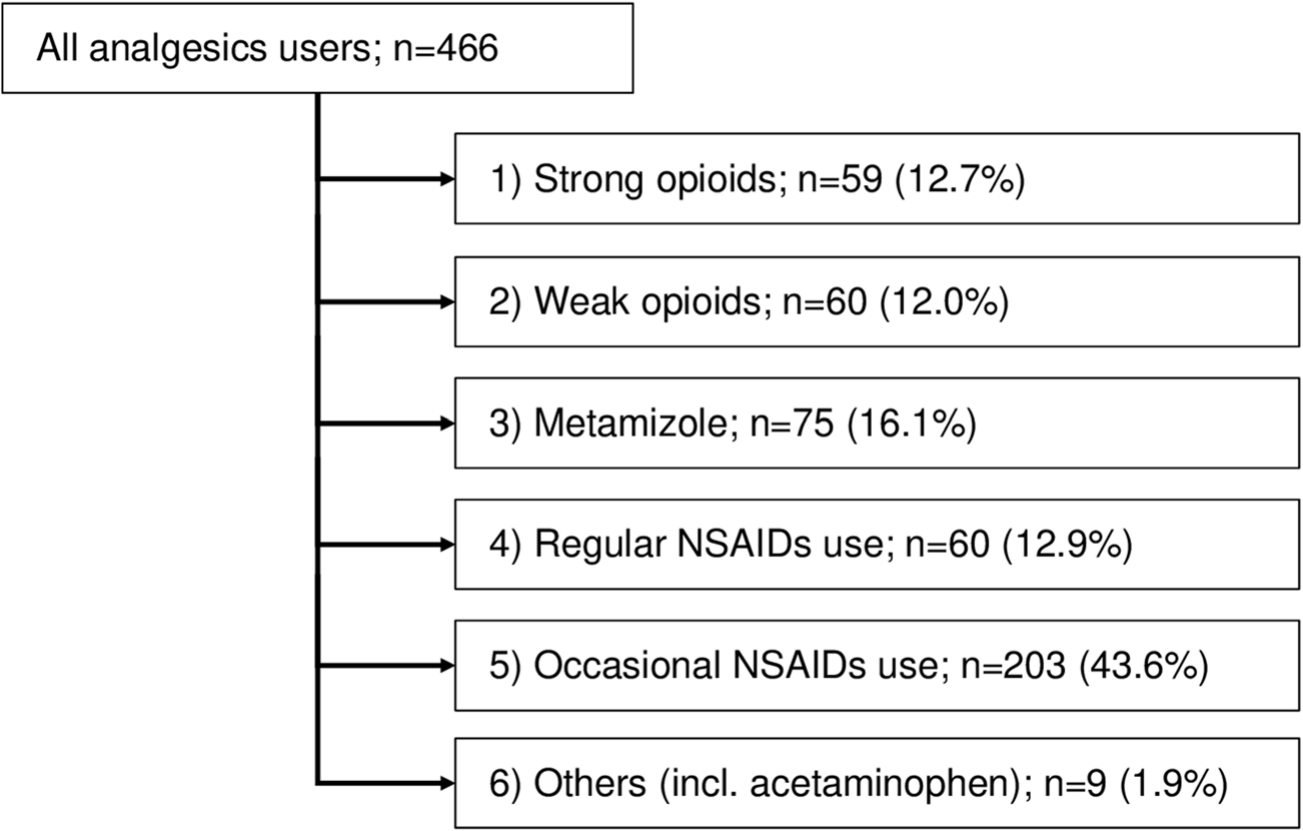
Allocation of an analgesics user to exclusive therapeutic groups in accordance with analgesics potency from strong opioids to acetaminophen

To create mutually exclusive groups, each of the 466 analgesics users was allocated into only one analgesics user group by its analgesics potency (Fig. 1). The percentages of users for the specific therapeutic groups shown in Fig. 1 were lower than those reported in Table 1 for some groups since concomitant use was excluded. This was especially true for the groups “adjuvant analgesics,” which were not listed in Fig. 1, and “others (incl. acetaminophen),” whose number was reduced to *n* = 9. The group “others” was not used in further analyses due to the low sample size.

**Fig. 2:**
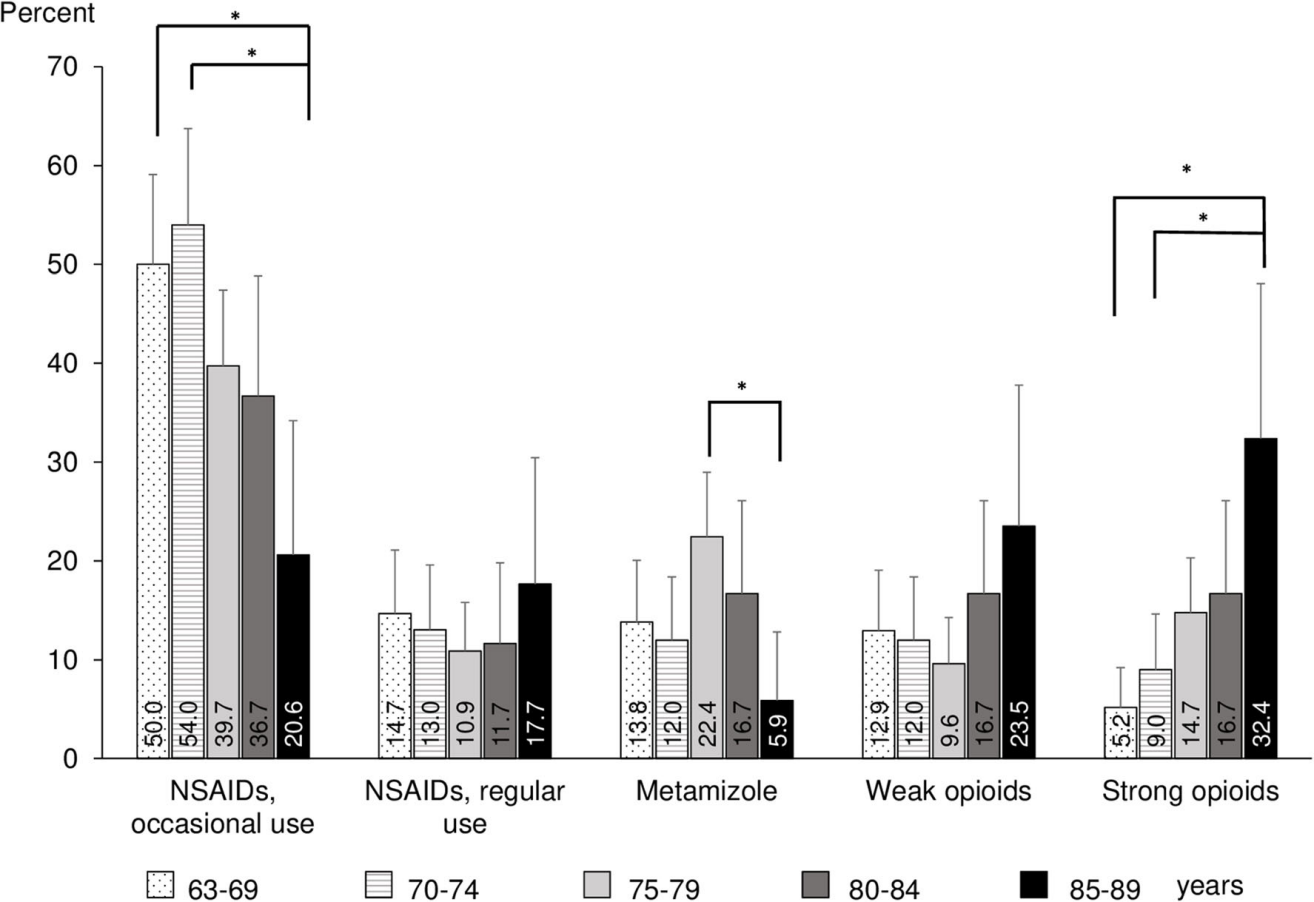
Utilization of different therapeutic analgesics groups across age groups. * statistically significant at *p* < .05

The prevalence of each of the five remaining therapeutic groups across age groups is shown in Fig. 2. In general, older patients tended to receive more strong opioids. The prevalence of opioids use was 5.2% in the group aged 63–69 years and was six times as high (32.4%) in the group aged 85–89 years. In contrast, older adults tended to use occasional NSAIDs less frequently. The prevalence of occasional NSAIDs use was 50% in the group aged 63–69 years and was much lower (20.6%) in the group aged 85–89 years. Nevertheless, apart from the oldest participants, aged 85–89, occasional NSAIDs use was the most popular choice among analgesics across all age groups.

**Fig. 3:**
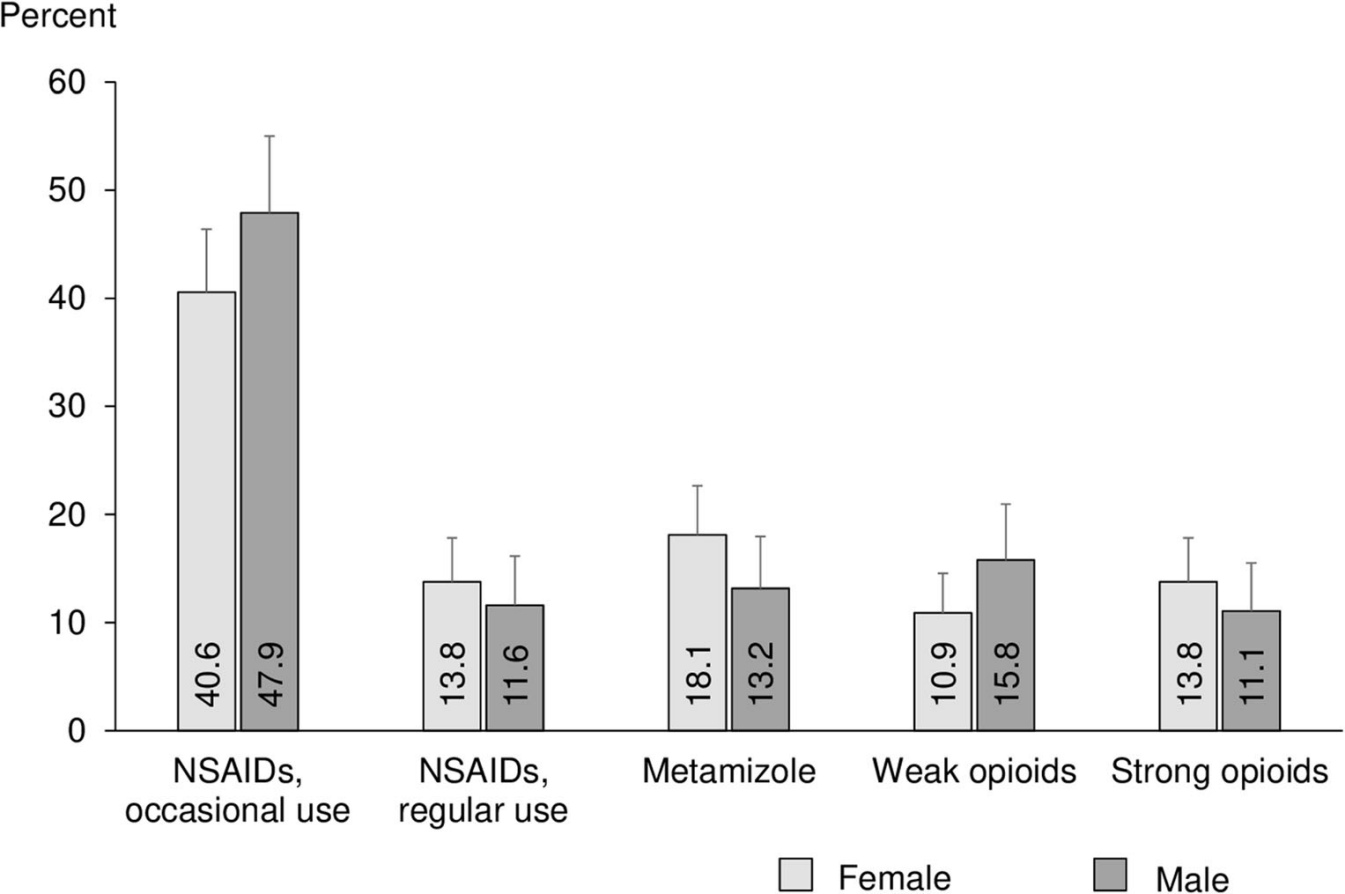
Utilization of different therapeutic analgesics groups in females and males

There were no statistically significant differences in analgesics utilization between male and female users (Fig. 3). However, we observed that metamizole was more frequently prescribed for females than males (18.1% vs. 13.2%), whereas weak opioids were less frequently prescribed for women than men (10.9% vs. 15.8%).

**Fig. 4:**
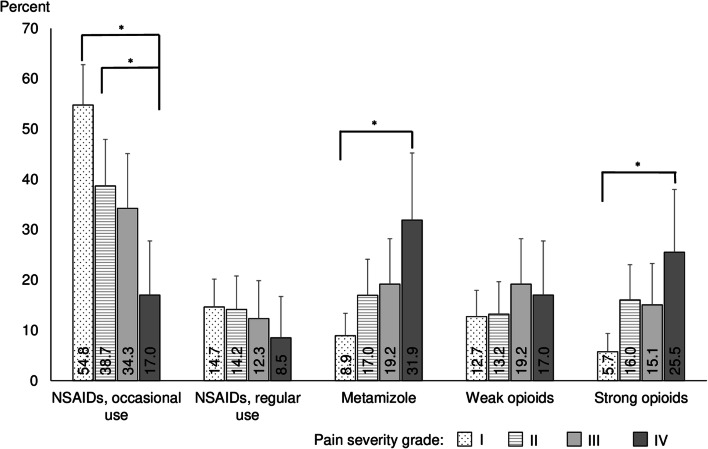
Utilization of different therapeutic analgesics groups according to pain severity grades I–IV. *statistically significant at *p* < .05

Figure 4 shows the prevalence of the therapeutic groups in study participants with different pain severities. Overall, both occasional and regular NSAIDs use prevalence got lower with higher pain severity of the participants. In contrast, metamizole and strong opioids use prevalence got higher in participants with higher pain severity grade (with the exception of strong opioids in pain severity grades II and III). There was no clear trend for weak opioids but their use was higher in pain severity grades III and IV than that in grades I and II.

**Fig. 5:**
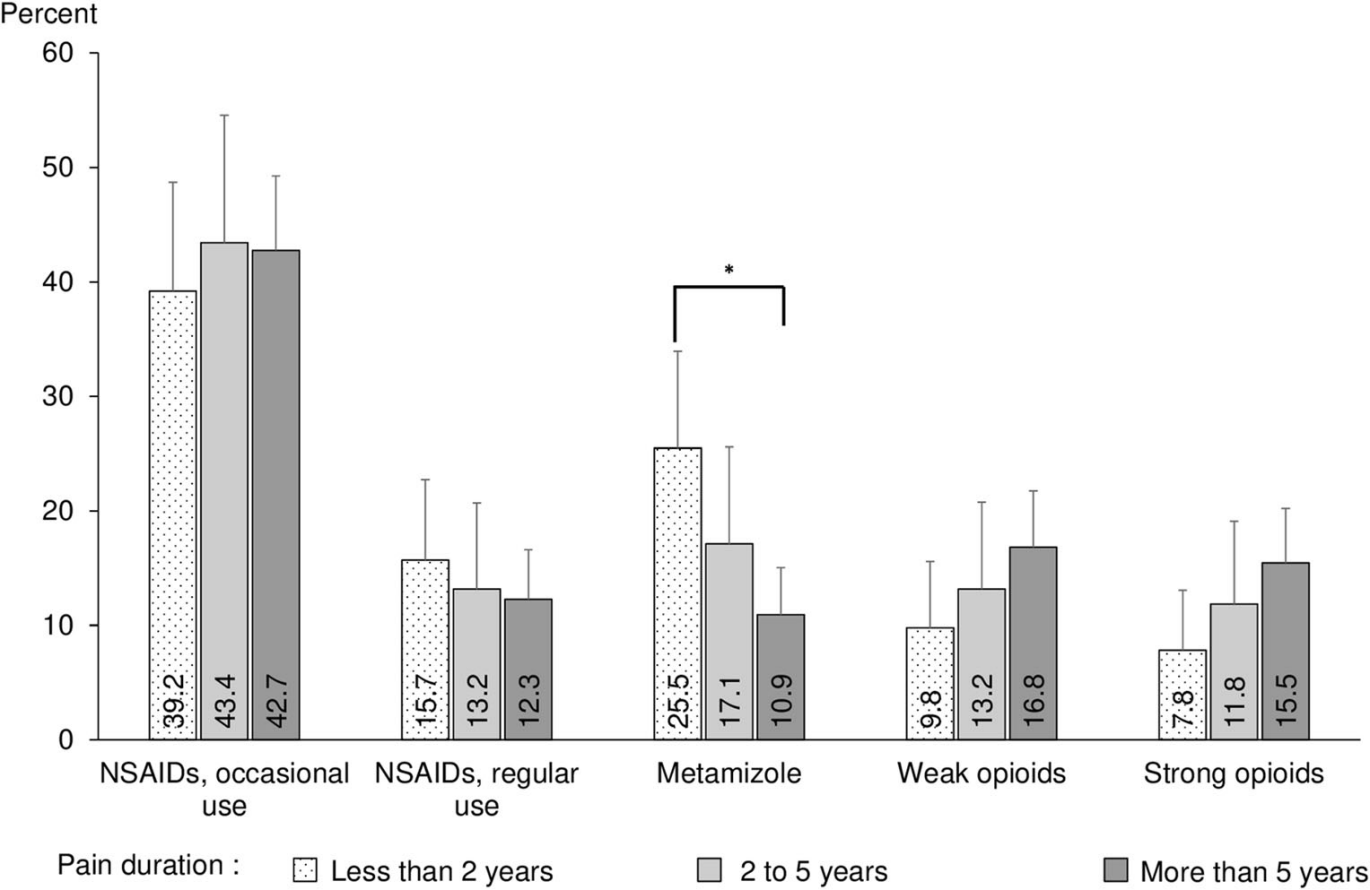
Utilization of different therapeutic analgesics groups according to duration of pain. *statistically significant at *p* < .05

Figure 5 shows the analgesics utilization patterns according to pain duration. No substantial differences were observed for both occasional and regular NSAIDs use. The lower the frequency of metamizole use, the longer the pain duration of the study participants, while frequency of use of both weak and strong opioids was higher accordingly.

**Fig. 6:**
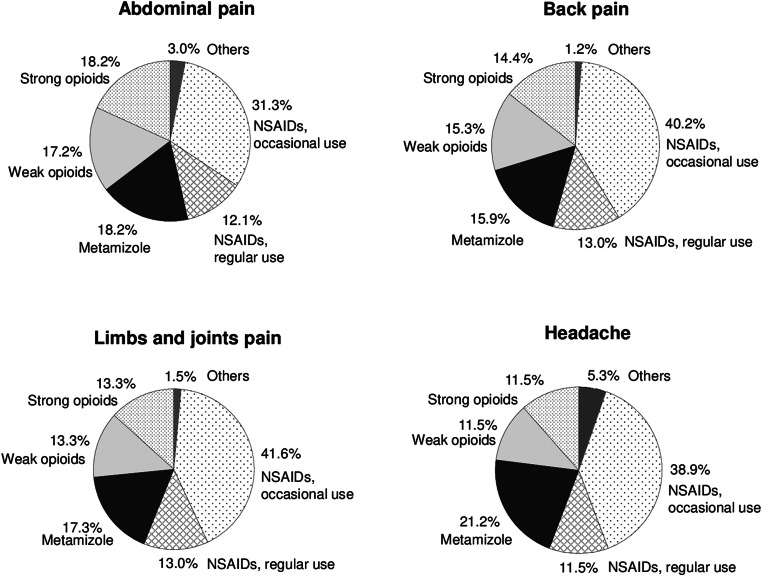
Utilization of different therapeutic analgesics groups by pain locations

Regarding pain locations, utilization of both strong and weak opioids was the highest for subjects reporting abdominal pain, followed by back pain, pain in the limbs or joints, and headache (Fig. 6). On the other hand, the use of occasional NSAIDs was lower among participants with abdominal pain than among patients with other pain locations.

### Factors associated with the utilization of opioids in a multivariate model

Table 3 shows the results of a multivariate logistic regression model for factors associated with opioids use (strong and weak opioids combined). Age ≥ 80 years, pain severity grades ≥ II, a pain duration of more than 5 years, abdominal pain, and back pain were statistically significantly associated with opioids utilization. Female sex, limb/joint pain, and headache might be inversely associated with opioids utilization (not statistically significant).

**Table 3:** Results of a multivariate model for factors potentially associated with a preference for opioids use among analgesics users (*n* = 431)

		*N*_total_^a^	*N*_opioids users_^a^ (%)	OR (95% CI)^b^
Age	63–69 years	108	21 (19.4)	Ref
	70–74 years	94	19 (20.2)	0.94 (0.45, 1.94)
	75–79 years	145	32 (22.1)	1.09 (0.57, 2.11)
	80–84 years	54	19 (35.2)	*2.23 (1.02, 4.90)*
	85–89 years	30	17 (56.7)	*4.89 (1.96, 12.50)*
Sex	Male	176	45 (25.6)	Ref
	Female	255	63 (24.7)	0.76 (0.46, 1.24)
Pain severity	0–I	213	35 (16.4)	Ref
	II	103	30 (29.1)	*1.97 (1.06, 3.66)*
	III	70	24 (34.3)	*2.20 (1.11, 4.32)*
	IV	45	19 (42.2)	*2.79 (1.26, 6.14)*
Pain duration	Less than 2 years	152	23 (15.1)	Ref
	2 years to 5 years	72	19 (26.4)	1.63 (0.76, 3.45)
	More than 5 years	207	66 (31.9)	*2.17 (1.23, 3.95)*
Pain location	Abdominal			
	No	341	77 (22.6)	Ref
	Yes	90	31 (34.4)	*1.88 (1.06, 3.30)*
	Back			
	No	104	13 (12.5)	Ref
	Yes	327	95 (29.1)	*2.15 (1.10, 4.47)*
	Limbs and joints			
	No	107	25 (23.4)	Ref
	Yes	324	83 (25.6)	0.72 (0.40, 1.32)
	Head			
	No	325	84 (25.8)	Ref
	Yes	106	24 (22.6)	0.66 (0.36, 1.16)

Statistically significant results are in italics

*N*_*total*_ total study population, *N*_*opiod users*_ number of opioids users, *OR* odds ratio, *CI* confidence interval, *Ref* reference category

^a^Complete case analysis conducted with *n* = 431 study participants of whom 108 were opioids users. Of those study participants who use any analgesic drug (*n* = 466), we excluded those who have missing values for any variables of the multivariate model (*n* = 35)

^b^Model contains all variables shown in the table

### Factors associated with the utilization of metamizole in a multivariate model

Table 4 shows the results of a multivariate logistic regression model for factors potentially associated with a choice of metamizole use over analgesics with similar or weaker analgesics potency. The age group from 75 to 79 years (compared with age 63–69 years) had statistically significantly increased odds for metamizole utilization but there was no clear pattern for other age groups. Pain severity grades ≥ II were statistically significantly associated with metamizole utilization and especially subjects with pain severity grade IV had strongly increased odds to be a metamizole user. Pain duration was statistically significantly inversely associated with metamizole utilization. Female sex and abdominal pain might also be associated with higher metamizole utilization (not statistically significant).

**Table 4:** Results of a multivariate model for factors potentially associated with a choice of metamizole use over analgesics with similar or weaker analgesics potency (i.e., opioid users are excluded, *n* = 323)

		*N*_total_^a^	*N*_metamizole users_^a^ (%)	OR (95% CI)^b^
Age	63–69 years	87	15 (17.2)	Ref
	70–74 years	75	12 (16.0)	0.74 (0.30, 1.79)
	75–79 years	113	34 (30.1)	*2.14 (1.03, 4.65)*
	80–84 years	35	8 (22.9)	1.64 (0.55, 4.72)
	85–89 years	13	2 (15.4)	0.59 (0.08, 2.81)
Sex	Male	131	24 (18.3)	Ref
	Female	192	47 (24.5)	1.48 (0.80, 2.79)
Pain severity	0-I	178	25 (14.0)	Ref
	II	73	18 (24.7)	*3.12 (1.42, 6.93)*
	III	46	14 (30.4)	*3.68 (1.53, 8.86)*
	IV	26	14 (53.9)	*13.10 (4.56, 40.01)*
Pain duration	Less than 2 years	129	36 (27.9)	Ref
	2 years to 5 years	53	11 (20.8)	*0.40 (0.16, 0.93)*
	More than 5 years	141	24 (17.0)	*0.32 (0.16, 0.62)*
Pain location	Abdominal			
	No	264	54 (20.5)	Ref
	Yes	59	17 (28.8)	1.87 (0.90, 3.80)
	Back			
	No	91	18 (19.8)	Ref
	Yes	232	53 (22.8)	0.82 (0.40, 1.69)
	Limbs and joints			
	No	82	14 (17.1)	Ref
	Yes	241	57 (23.7)	1.07 (0.51, 2.32)
	Head			
	No	241	49 (20.3)	Ref
	Yes	82	22 (26.8)	1.18 (0.59, 2.29)

Statistically significant results are in italics

*N*_*total*_ total study population excluding opioid users, *N*_*metamizole users*_ number of metamizole users, *OR* odds ratio, *CI* confidence interval, *Ref* reference category

^a^Complete case analysis conducted with *n* = 323 study participants and 71 metamizole users. Of those study participants who use any analgesics drug (*n* = 466), we excluded opioids users (*n* = 119) and those who have missing values for any variables of the multivariate model (*n* = 24)

^b^Model contains all variables shown in the table

## Discussion and conclusions

### Overall analgesics use and pain intensity

The overall prevalence of analgesics use in our study was in line with those observed in two previous studies with community-dwelling older adults from Germany [13, 17]. Compared with the patients with multiple conditions in the study of Freytag et al., our participants were healthier, which explained the lower prevalence (22.9% vs. 36.7%). The study of Sarganas et al. reported a lower prevalence of 19.5% in study participants aged 65 years or older [13]. However, this study only focused on five commonly taken analgesics (aspirin, diclofenac, ibuprofen, acetaminophen, and naproxen), which explains the difference.

Comparing pain severity across studies is challenging due to the varying instruments used. To the best of our knowledge, there are no previous studies conducted in the general older German population using the Chronic Pain Grade [22]. Hauser et al. [4] applied this instrument to middle-aged German adults (mean age, 49.7 years) and noted prevalence of high-intensity or disabling pain of 15.6%. Participants in our study were older (mean age, 74.5 years), and thus had a higher prevalence of 25.0% because various chronic disorders associated with pain often accumulate at higher age. Accordingly, increasing age and the frequency of use of analgesics were associated with each other in our cross-sectional survey, which has also been reported previously [13, 28].

Half (50.5%) of those being treated with analgesics reported that they still had high-intensity or disabling pain. This surprisingly high proportion is in accordance with data from a pan-European survey. In this survey, 40% of those with long-lasting recurring pain reported that they were generally not satisfied with the efficacy of their treatment, and 64% said that their pain medication was currently not sufficient [5].

### Utilization patterns for therapeutic groups of analgesics

#### NSAIDs

As in our study, studies from Norway and Spain observed that NSAIDs use decreased with age [11, 29]. This complies with guidelines recommending avoiding NSAIDs for pain management in older adults whenever possible, due to high concerns of gastrointestinal hemorrhage, cardiovascular events, and renal impairment [7, 30]. However, it should be noted that 15.9% of our population still used NSAIDs regularly (12.9% as primary pain therapy and 3% in addition to opioids or metamizole). These numbers are concerning, giving NSAIDs’ negative benefit–risk profile in older adults. In the FORTA (Fit for the Aged) list, experts from Germany, Austria, and Switzerland rated long-term NSAIDs use as inappropriate for older adults and recommended finding alternatives [30].

#### Metamizole

An alternative to NSAIDs is metamizole, which is popular in Germany and most European countries [31]. In few countries, however, metamizole is not available on the market due to the rare but potentially fatal adverse event of agranulocytosis [9, 14, 15, 32]. Metamizole has at least a potency similar to most NSAIDs [25], and when used short term, it is also the safer choice [33, 34]. The risks and benefits of metamizole for long-term use (longer than 2 weeks), however, are still not well studied [16]. In Germany, its indications nowadays are restricted for treatment of acute severe pain after injury or surgery, colic or cancer pain, other acute or chronic severe pain when other drugs are not indicated, and high fever that does not respond to other therapies [35]. Nevertheless, in the FORTA list, metamizole is recommended for the management of chronic pain in older people if acetaminophen is not potent enough [30]. In our study, the odds for metamizole prescriptions were independent of age (with only one irrelevant exception), sex, and pain location. However, there was a clear positive correlation with pain severity (likely because it is only licensed for severe pain conditions) and a negative correlation with pain duration (maybe because of the lack of safety data for long-term use [16]).

#### Opioids

In contrast to NSAIDs, the prevalence of opioids use was higher in older than in younger age groups (especially in the age group 80–89 years). A similar trend was seen in a study in Germany, where the prevalence of high-potency opioids use was the highest in the group aged 80 years and older [36]. In the USA, the prevalence of long-term opioids use was also much higher among older (≥ 65 years) women and men [37]. Besides age, higher pain severity and longer pain duration were associated with opioids use in our study. It is plausible that the stronger the pain is and the longer it persists, possibly due to treatment failure of other analgesics, the more likely opioids prescriptions are given. This might especially be true at an advanced age of 80 years and older when diseases accumulate and cause chronic pain.

Some opioids are recommended by the FORTA list for use in older adults (buprenorphine, oxycodone, hydromorphone), while others are not (tilidine/naloxone, oxycodone/naloxone, and morphine). In older patients, physicians are most concerned about opioids’ effect on mental status (sedation and cognitive impairment) and balance (increased risk of falls). Therefore, older patients who receive opioids for the first time need to be monitored closely [38]. Patients, on the other hand, are worried about the risk of addiction [5], or are frequently troubled by constipation [39, 40].

These justified concerns surrounding opioids, which are prevalent on both sides of patients and prescribers, may explain in part the poor pain control in our study population. Compared with other European countries, Germany ranks first in terms of overall narcotic drug consumption but utilization of high-potency opioids (e.g., morphine, hydromorphone, and buprenorphine) is lower than that in other European countries (International Narcotics Control Board, 2018). Nonetheless, better pain control could likely be obtained with more prescriptions of strong opioids. Nearly half of the participants receiving analgesics still suffered from high-intensity or disabling pain (grades II–IV). For these patients, almost one-third were prescribed metamizole (31.9%); and one-fourth (25.5%), NSAIDs.

## Strengths and limitations

This study has some limitations. First, our results are based on a sample of German, community-dwelling adults aged 63–89 years and cannot be generalized to other countries, age groups, or nursing home residents. Second, participants of the ESTHER study who agreed to take part in the home visit were healthier than those who only sent back the participant’s questionnaire and people with cognitive problems or severe frailty are likely underrepresented [41]. Therefore, all reported prevalence estimates affected by morbidity (e.g., chronic pain prevalence, pain severity, and use of analgesics, especially opioids) could be underestimated. Third, we did not assess the indication for the drugs; thus, it was not possible to state whether antidepressants or anticonvulsants were prescribed as adjuvant analgesics. We only counted these drugs as adjuvant analgesics when they were combined with other pain medications and did not put focus on them in the analyses. Fourth, we had no information on the history of attempts with other analgesics in the past, which may have been prescribed due to treatment failure or adverse events. Fifth, we did not consider co-medications or co-morbidities that potentially prohibit NSAIDs (e.g., contraindicated drug–drug interactions, previous peptic ulcer bleedings, or severe heart failure) or opioids use (e.g., severe broncho-pulmonary obstructive disease, myasthenia gravis).

Our study also has strengths. The “Brown Bag” method ensured a complete assessment of all used drugs, including over-the-counter preparations, numerous of which are NSAIDs. Intentional non-adherence was low, affecting only 1.2% of all recorded drugs [42]. This low number may be explained by the study protocol, which said that study physicians shall ask participants to show only the medications they currently have at home and use regularly or as needed. Furthermore, we applied the validated German version of the Chronic Pain Grade questionnaire [21], which allowed interesting clinical insights into the success of pain management for older German adults.

## Conclusions

In this study of 2038 older Germans with a mean age of 74.5 years, one in two reported pain in the last 4 weeks and 1 out of 4 suffered from either high-intensity pain or disabling pain. However, less than 1 in 4 used analgesics and about half of those who used analgesics still had high-intensity pain or disabling pain. The insufficient pain control in our study population could be a sign for undertreatment with opioids in older German adults. However, withholding these drugs from older adults as long-term therapy can also be justified because of adverse events or treatment failure. Studies comparing the effectiveness and safety of different analgesics in long-term therapy of chronic pain for older adults are needed.

## Electronic supplementary material

ESM 1 (PDF 377 kb)
