# Supplementary material for: Pain severity and analgesics use in the community-dwelling older population: a drug utilization study from Germany
Source: Eur J Clin Pharmacol. 2020 Jul 10;76(12):1695–707. doi: 10.1007/s00228-020-02954-5 (PMC7661425; doi:10.1007/s00228-020-02954-5)
Supplement: Supplementary file 1 — (PDF 377 kb) [file 228_2020_2954_MOESM1_ESM.pdf]

Pain severity and analgesics use in the community-dwelling older population. A drug utilization study from Germany

Thi Ngoc Mai Nguyen, MSc[1,2]; Dana Clarissa Laetsch, PhD[1]; Li-Ju Chen, MSc[1,2]; Walter Emil Haefeli MD[3]; Andreas D Meid, PhD[3]; Hermann Brenner, MD[1,2]; Ben Schöttker, PhD[1,2‡]

[1]Division of Clinical Epidemiology and Aging Research, German Cancer Research Center (DKFZ), Heidelberg, Germany

[2]Network Aging Research, University of Heidelberg, Heidelberg, Germany

[3]Department of Clinical Pharmacology and Pharmacoepidemiology, Heidelberg University Hospital, Heidelberg, Germany

‡Correspondence:

Ben Schöttker, PhD, MPH, MSc

Division of Clinical Epidemiology and Aging Research at the German Cancer Research Center (DKFZ) and Network Aging Research of Heidelberg University (NAR), Im Neuenheimer Feld 581, 69120 Heidelberg, Germany, Telephone: +49 6221 42-1355, Fax: +49 6221 42-1302, E-mail: b.schoettker@dkfz.de

Supplementary Material

Table S1 Selected ATC codes for therapeutic analgesics groups

Strong opioids[a]	Weak opioids[a]	Metamizole	NSAIDs[b]	Others	Adjuvants[c]
N02AA01	N02AA08	N02BB	M01A	A03D	N03AF01
N02AA03	N02AA58		M01BA	N02BE01	N03AX09
N02AA05	N02AA59		N02BA	N02BE03	N03AX12
N02AA25	N02AA69			N02BE04	N03AX16
N02AA51	N02AA79			N02BE05	N06AA01
N02AA53	N02AJ01			N02BE53	N06AA02
N02AA55	N02AJ02			N02BE54	N06AA04
N02AA56	N02AJ03			N02BE71	N06AA06
N02AB03	N02AJ05			N02BE73	N06AA09
N02AE01	N02AJ06			N02BE74	N06AA10
N02AG01	N02AJ07			N02BG	N06AA21
N02AG04	N02AJ08			N02C	N06AB03
N02AJ17	N02AJ09				N06AB04
N02AJ18	N02AJ13				N06AB05
N02AJ19	N02AJ14				N06AX16
N02AX06	N02AJ15				N06AX21
	N02AX01				
	N02AX02				
	N02AX51				

Notes: Preparations licensed only for the treatment of colds (e.g., codeine preparation ATC codes R05DA04 or R05DA14) are not included. ATC codes without any preparations available on the German market are not listed.
[a] Combinations with opioids are included in opioid groups only.
[b] Combinations with NSAIDs other than opioids are included in NSAIDs groups only
Abbreviations: ATC, Anatomical Therapeutic Chemical Classification
[c] Only adjuvants mentioned in the German guideline for treatment of non-specific lower back pain (https://www.leitlinien.de/nvl/html/kreuzschmerz/kapitel-6) or the German guideline for the therapy of neuropathic pain (https://www.dgn.org/images/red_leitlinien/LL_2008/archiv/ll08kap_064.pdf) as licensed or recommended for off-label use for the long-term treatment of either chronic pain or neuropathic pain are included.
